# Externalities from Alcohol Consumption in the 2005 US National Alcohol Survey: Implications for Policy

**DOI:** 10.3390/ijerph6123205

**Published:** 2009-12-11

**Authors:** Thomas K. Greenfield, Yu Ye, William Kerr, Jason Bond, Jürgen Rehm, Norman Giesbrecht

**Affiliations:** 1 Alcohol Research Group, 6475 Christie Avenue, Suite 400, Emeryville, CA 94608, USA; E-Mails: yye@arg.org (Y.Y.); wkerr@arg.org (W.K.); jbond@arg.org (J.B.); 2 Clinical Services Research Training Program, Department of Psychiatry, University of California, San Francisco, 401 Parnassus Avenue, San Francisco, CA 94143, USA; 3 Centre for Addiction and Mental Health, 33 Russell Street, Toronto, Ontario, M5S 2S1, Canada; E-Mails: jtrehm@aol.com (J.R.); Norman_Giesbrecht@camh.net (N.G.); 4 Dalla Lana School of Public Health, University of Toronto, 155 College Street, Toronto, Ontario, M5T 3M7, Canada

**Keywords:** externalities, alcohol consumption, heavy drinking, population survey, impact, policy, economics, cost, environment, US

## Abstract

A subsample (*n* = 2,550) of the 2005 US National Alcohol Survey of adults was used to estimate prevalence and correlates of six externalities from alcohol abuse––family problems, assaults, accompanying intoxicated driver, vehicular accident, financial problems and vandalized property––all from another’s drinking. On a lifetime basis, 60% reported externalities, with a lower 12-month rate (9%). Women reported more family/marital and financial impacts and men more assaults, accompanying drunk drivers, and accidents. Being unmarried, older, white and ever having monthly heavy drinking or alcohol problems was associated with more alcohol externalities. Publicizing external costs of drinking could elevate political will for effective alcohol controls.

## 1. Introduction

Externalities from alcohol consumption may be considered to be the burdens that fall upon others rather than the individuals choosing to drink. Such externalities may be inflicted upon other individuals or upon society at large––for example when a person’s property is destroyed by someone else who is drunk, or when indigents injured by their drinking must be cared for in an emergency department or trauma center, with costs transferred to the public. From an economic perspective, externalities are important in that they are likely to fall outside the individual’s utility estimation in making the choice to drink in the way they do. For example Pogue and Sgontz [1] note: “An individual’s demand for alcohol will reflect the extent to which he perceives and takes account of internal abuse costs” (p. 235). To Pogue and Sgontz, internal costs include, for example, things like increased medical expenses, lost income, and the personal pain and distress associated with excessive use. Of course, perceptions of risks of even such internal harms may be distorted but nonetheless a person’s own costs and benefits are in principle weighed’ in choosing to drink a given amount. While altruism or self interest in others’ good (e.g., the costs and benefits of drinking to one’s immediate family) could in principle enter one’s utility set, generally the utility appraisal is taken by economists as limited to self. Others’ harms from a person’s drinking are typically taken as external to the individual’s drinking decisions [2,3]. “External abuse costs…take a number of forms, mainly injury to others and damage to their property” [1] (p. 235, where a footnote calls attention to the fact that “the financing of health care and the pricing of insurance do not at present confront consumers with the full costs of their actions…”).

Increasing alcohol excise taxes or otherwise raising prices or decreasing availability of beverage alcohol are among the policy measures that have been found particularly effective in reducing ethanol consumption and alcohol-related problems; see for current review[4]. Cook and Moore [5] note that this “set of findings is relevant for policy purposes because alcohol abuse imposes large ‘external’ costs on others” (p. 120). Even with their rather restricted view of externalities, as cited earlier, Pogue and Sgontz [1] found, using various plausible assumptions, that of the total US national costs of alcohol abuse of $116.7 billion in 1993 [6], $26.1 billion or 22.4%, could be considered external, the remainder being internal.

The aim of this paper is to contribute to the assessment of several kinds of externalities, those reported by US national survey respondents to stem from others’ drinking. We also examine the extent to which such externalities (problems from others’ drinking) are associated with the respondent’s demography and their own alcohol use patterns and alcohol-related problems. The latter is important because drinkers, by seeking out environments frequented by other drinkers, could receive a larger than average share of the “fall out” from others’ drunken behavior.

For many externalities from alcohol consumption, data are available to allow estimation of certain costs to society, at least under various assumptions. Both archival records and self reports each have their own deficiencies and may not always agree—e.g., for alcohol involved vehicular offences, see [7]—nevertheless some meaningful estimates are possible. For example, on a national basis, criminal justice system costs [8] and those public costs for health care resulting from harmful drinking but not borne by the individual have been estimated for the US [9–14] and some Canadian provinces [15]. Efforts have been made to standardize approaches to making such cost estimates [16]. Fairly good data are available on collateral mortalities arising from drunk driving, e.g., to passengers, pedestrians or those in other vehicles [17–19]. Something is known about victimization by physical assault [20] and other crimes such as burglary, theft and rape by perpetrators reported to have been drinking [21–23]. However, only if alcohol were causally linked would such problems truly be externalities.

There are decades of epidemiological surveys in which respondents are asked about *their own* problems [24]. Despite issues of potential biases inherent in self reporting, theses assessments are obviously vital for identifying hazardous and harmful patterns of drinking that are linked to individual harms and the trends in individuals’ drinking levels and their own harms [25]. However, there has been little study of respondents’ reports of harms by other drinkers [26]. Therefore, much less is known about instances where individuals feel they have been harmed by others’ drinking, particularly the more common and less severe forms of risks they may be exposed to associated with *others’* drinking (such as when being a passenger of a drunk driver) or reporting actual harms associated with someone else’s drinking. Only a few surveys have asked about such risks and harms from others’ drinking. The 1989 Canadian National Alcohol and Other Drugs Survey (Canadian AODS) [27] included 10 questions of this sort and assessed occurrence both on a lifetime basis and in the prior 12 months. Included were being insulted or humiliated, disturbed by loud parties, having been a passenger with a drunk driver, experiencing arguments, family problems, physical assault, having lost friends, having property vandalized, being in an accident or having financial problems due to someone else’s drinking. Being insulted/humiliated and disturbed by loud parties were most common (close to 50% each on a lifetime basis and 21 and 26%, respectively, during the prior year), as was being a passenger with a drunk driver (37% lifetime, 10% 12 months). Family problems and assaults due to another’s drinking were common also (21% and 20% lifetime, and 8% and 7% 12 months, respectively. Even the least reported items, been in an accident and financial problems (7% and 5% lifetime, and 1% and 2% 12 months, respectively) were reported by about one in 20 on a lifetime basis. Therefore, cumulatively, almost four of five (78%) Canadians had experienced a problem attributed to have resulted from someone else’s drinking at some time in their lives, 45% during the year preceding the survey. Based on bivariate analyses, men (47%) were slightly more likely than women (43%) to report such problems on a 12-months basis (also lifetime). Men were a bit more likely than women to have ever been a passenger with a drunk driver (39% *versus* 35%, respectively) and to have been physically assaulted (24% *versus* 16%, respectively). However, over their lives women were twice as likely as men to report family problems (28% *versus* 14%, respectively), and financial problems (7% and 3%, respectively), from someone else’s drinking. These gender differences accord with the notion that heavier drinking by men impinges on their families. Rates of suffering these externalities of drinking in the prior year peaked at ages 20–24 and declined thereafter. Education and income had few clear relationships. Current drinkers were more likely to encounter problems from others’ drinking in the last year than former drinkers or lifetime abstainers. Both volume and frequency of heavy drinking were positively related to reporting problems from others’ drinking. Also, 73% of those reporting an alcohol-related problem of their own last year also experienced a problem from others’ drinking, whereas the prevalence of externalities was 45% in the absence of having any of one’s own alcohol problems [27, pp. 232–237].

In Australia, the 2001 National Drug Strategy Household Survey included some items on victimization related to alcohol or other drugs, namely suffering verbal abuse, physical abuse or “being put in fear” by another’s drinking. “Australians aged 14 and over were more than twice as likely to be victims of alcohol-related incidents [28] than incidents related to other drugs” [29, p. 39]. Males (29.2%) were more prone than females (23.8%) to report verbal abuse and physical abuse (5.8% *versus* 3.9%, respectively) but women were more likely than men to have been ‘put in fear’ by a person under the influence of alcohol (15.6% *versus* 11.8%, respectively).

Only one US study was found that included reports of problems from others’ drinking, specifically a Marin County, California, general population survey conducted in 1990 (*n* = 1,985). Using five items taken from the above Canadian national survey, Jones and Greenfield [30] found that 71% of adults reported ever being affected by someone else’s drinking (19% 12 months). The most reported externalities were being a passenger with a drunk driver or having marital or family problems (53% and 36% lifetime, and 9% each, 12 months, respectively). Physical abuse due to another’s drinking was reported by 30% on a lifetime, and 6% on a 12-month basis. People with negative experiences from others’ drinking tended to be younger (18–24), single (never married), and unemployed and, unlike Canada, women were more likely than men to report any such harms from others’ drinking [30, pp. 16–17].

The externalities measure, a composite of the harm from others’ drinking, termed *Trouble from others’ drinking* was used earlier [31] as a predictor of policy opinions in prior research using the 2000 National Alcohol Survey (NAS). Hypothetically, perceived harm from others’ drinking could be very important for shaping ones’ attitude toward the need for alcohol control policies. Differing from the present study, the earlier *Trouble from Others* scale score was the summation of eight dichotomous items measuring unpleasant experiences with other people’s drinking problems during the last 12 months, the six items focused on here—family or marriage difficulties, being a passenger of a drunk driver, having a motor vehicle accident involving someone else’s drinking, being hit or assaulted, having financial problems, property vandalized—but additionally, two further items: suggesting that others seek help for alcohol problems or assisting them to do so. The 8-item trouble from others’ drinking scale had adequate internal consistency (alpha = 0.61) for research purposes. This externalities scale was found to significantly predict each of four alcohol policy opinion factors [31]. The four factors were *Control Measures* like taxation and reduced access, *Alcohol Interventions* like favoring more alcohol prevention and responsible beverage service programs, *Warning Labels* on containers and advertisements, and *Treatment Access* like insurance coverage and free access [32]. Because of the balloting schemes in the 2005 NAS, the policy items and externalities measures appear in separate ballots, so policy measures cannot be used in the present analysis of the 2005 NAS data.

## 2. Methods

### 2.1. Sample

The 11^th^ 2005 National Alcohol Survey (NAS 11) was conducted for the Alcohol Research Group by DataStat, Inc., of Ann Arbor, Michigan, between November, 2004 and July, 2006 and yielded 6,919 respondents with completed interviews. The sampling frame was the 50 US states plus Washington District of Columbia (DC), and interviews in all these jurisdictions were conducted using a modified list-assisted Random Digit Dial (RDD) method and computer assisted telephone interviewing (CATI). Reverse directory look up allowed prior mailings to approximately half the respondents. Additionally, multiple, largely unlimited callbacks and extensive refusal conversion attempts were used to minimize non-response. Bilingual interviewers conducted interviews in Spanish when necessary or requested. In each household, a random respondent was chosen using the last birthday technique (selecting the individual listed as having the most recent birthday). The response rate based on cooperation was 56% for the 2005 NAS (similar to that obtained for the 2000 NAS). This rate is not untypical of telephone surveys [33] and it should be noted that non-response in telephone surveys is generally deemed less biasing than in face-to-face surveys because hang-ups, the largest basis for non-response, often occur before the topic of the survey has even been broached. Furthermore, comparison between large telephone (1990 WL) and in-person (1990 NAS) surveys (with lower and higher response rates, respectively, but conducted in the same year) did not revealed significant differences in national estimates of alcohol consumption [34] or major differences in alcohol-related harms. Greenfield *et al*. [35] discusses further non-response studies conducted in the 2000 NAS that again found little evidence of effects on drinking variables due to differential response rates in random subsamples, suggesting that lower response rates seen in typical telephone surveys, compared to those using in-person interviews, may not introduce substantial additional bias.

Given the extensive length of the survey instrument, while all respondents were asked questions like demographics and drinking characteristics, questions on externalities and some other question sections were only administered to randomly-selected subgroups through a balloting process, which in this instance represented approximately 37% of the total sample (*n* = 2,550). Of the included group, 54% of respondents were women. The percentages of those aged 18–29, 30–49, and 50+ were 20%, 40%, and 40%, respectively. Like its predecessors, the 2005 NAS oversampled black and Hispanic individuals and the balloting took advantage of these oversamples. The subgroup for analysis was composed of 32% Hispanics, 20% black non-Hispanics, 43% whites and 5% other ethnicities or races (unweighted percentages). These gender, age and racial/ethnic subgroup distributions for the subgroup were very similar to the total sample of 2005 NAS excepting in the lower proportion of whites and others, groups the balloting process in this case sampled these with a lower sampling fraction. This was done to assure adequate power for the planned ethnic group comparisons. For the analyses reported here, weighting was used to adjust the analysis subsample to the general population percentages. First, weighting took account of probabilities of selection based on the number of the independent land telephone lines in the household and the number of household adults. Weighting factors also included geographic region, age, gender and ethnicity, adjusting for non-response and the ethnic minority oversampling. For the main analyses reported here we used the Survey commands in Stata, Release 9 [36] to adjust the standard errors based on the sampling design, including ethnic/racial strata and the post-stratification weights. Table 1 provides the demographic distributions in the sample used for analysis (unweighted *n*’s, weighted percents).

### 2.2. Measures

Included are six externality items taken from the 1989 Canadian AODS [27]. The six items are given verbatim in Table 2. The preamble instruction read: “The next few questions concern your experiences with other people’s drinking problems. Have you ever (READ ITEM).” An example item is “had family problems or marriage difficulties due to someone else’s drinking?” Following the lifetime query for each item, respondents were asked “Was this during the last 12 months?”

Demographic items included: gender, age (categorized for this analysis: 18–29, 30–49 and 50+), a variable indicating the major ethnic groups (white, Hispanic, black non-Hispanic, and other groups), married *versus* other marital status, employed *versus* not, education (high school graduate or less, some college and college graduate), income (less than $20,000, $20,000–60,000, $60,000+ and Missing–amounting to 13%) and whether or not there were children under 18 living with the respondent.

Several drinking status variables were used, depending on the analysis. For multivariate logistic regression analyses predicting lifetime report of 2 or more (2+) externalities, a Lifetime Drinker Status variable was constructed including the following categories: lifetime abstainer, never drank five or more (5+) drinks in a day more than 11 times a year, reported 5+ at least monthly at some time (during teens and, if age relevant, in 20s, 30s, or currently), and lifetime alcohol-related problems. For this purpose the problem measure was whether or not on a lifetime basis the respondent reported either (or both) 2+ of 15 tangible alcohol-related consequences or 3+ alcohol dependence symptoms from seven separate DSM-IV domains [37] see further below. This Lifetime Drinker Status measure (excepting the lifetime abstention category) applies to current and ex-drinkers.

For descriptive analyses a different classification was used involving 10 categories. The first two were Lifetime Abstention and Ex-Drinker. Remaining categories involved volume and pattern measures and were empirically generated using a segmentation analysis designed to identify meaningfully distinct drinking patterns (in terms of predicting externalities). The segmentation analysis was implemented by Answer Tree 3.0 [38], an update of CHAID (CHi-squared Automatic Interaction Detection) [39]. First, a 10-level average alcohol consumption measure (number of drinks per day) was constructed based on the Graduated Frequency (GF) measure [40,41]. Following a question on maximum number of drinks consumed [35], frequencies of drinking at a descending series of quantity levels is assessed. The GF measure has been validated against daily diary measures [42,43] and captures heavy drinking episodes better than QF measures based only on usual quantities and frequencies [44]. Additional pattern measures included in the segmentation analysis were frequencies of drinking 5+, 8+ and 12+ drinks (all categorized as Never, Yearly but less than monthly, Monthly, Weekly and More Often) and also based on GF data. First, the segmentation analysis reduced the useful volume levels to five empirically distinct levels: One drink/month or less; 1+ to 2 drinks/month; 2+ drinks/month to 2 drinks/week; 2+ drinks/week to 4 drinks/day; and Over 4 drinks/day. Next, heavy drinking pattern measures were introduced. The two lowest volume categories could not be empirically split by pattern; the <2 drinks/week category was split by the pattern Never 5+ *vs.* 5+ at least yearly; the <4 drinks/day category was split by Never 5+ monthly *vs.* 5+ at least monthly; and the Over 4+ drinks volume category was split by the pattern measure Never 12+ monthly *vs.* 12+ drinks at least monthly. For details of a similar CHAID analysis, see Greenfield, *et al*. [45,46].

Several Problem Drinking variables were used, two involving symptoms generally defining alcohol use disorders. First, similar to alcohol abuse, a 15-item scale assessed Consequences in terms of social or health problems, based on positive responses to two or more of 15 items involving job-workplace problems (3 items), trouble with the law (3 items), aggression (4 items), social and health problems (3 items) and accidents (2 items) [47,48]. Second, we used an Alcohol Dependence scale involving 17 items assessing aspects in each of 7 areas reflecting the symptom definitions for each of the domains in the DSM-IV alcohol dependence [49,50]. Consistent with the DSM-IV criteria, at least one positive item is needed from each of three domains (out of seven) to meet the criterion for Alcohol Dependence. Unlike DSM-IV diagnosis, though, no concurrency within a 2 week period was required. Finally, we included in correlation analyses a life-area harms indicator with one or more of six areas reported to have been harmed by the respondent’s own drinking (lifetime and 12 months). Life harms assessed included friendships and social life, outlook on life, home life or marriage, financial position, work and employment opportunities, and health.

## 3. Results

**Figure 1.**
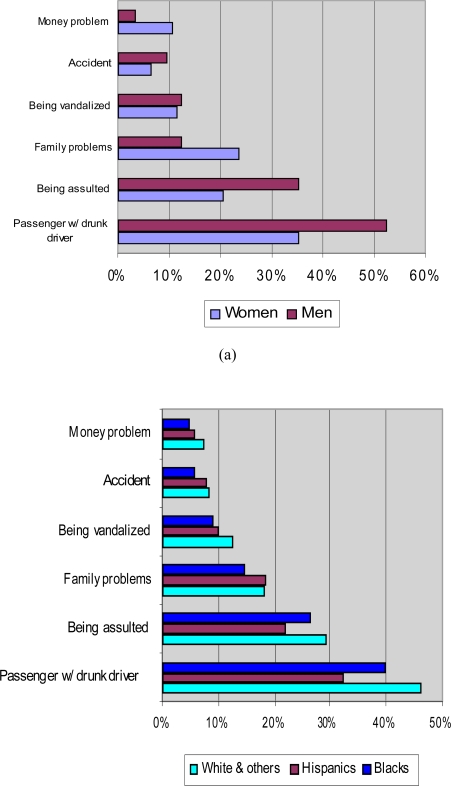
Prevalence of Individual Lifetime Externalities (a) by Gender; (b) by Ethnicity.

Table 2 shows the population prevalence of ever experiencing each of the six individual externality items “due to someone else’s drinking” on a lifetime basis and within last 12 months. On the lifetime basis, *being a passenger with a drunk driver* was reported by the largest proportion of the general population (44%), followed by *being assaulted* (28%). Overall, about 60% of the US population reported at least one problem by others’ drinking problems in their lifetime and 34% reported two or more such problems. In comparison, externalities in the last 12 months were reported by many fewer individuals, with about 9% experiencing at least one problem from others’ drinking. Considering gender differences on the lifetime basis, men and women varied on their exposure to specific externalities, with women almost twice as likely as men to have reported experiencing *family or marital problems* (24% *vs.* 13%) and much more likely to have experiences *financial problems* attributed to another’s drinking (11% *vs.* 4%), as shown in Figure 1a. With regard to ethnic/racial differences, whites and other ethnic/racial groups reported a higher rate of 2+ externalities over their lives (36%) than non-Hispanic blacks (28%) and Hispanics (26%). This pattern held for most of individual externality items (see Figure 1b).

For the multivariate logistic regression analyses predicting occurrence of externalities, demographic and drinking status variables were entered simultaneously to predict the likelihood of reporting 2+ externalities on the lifetime basis and 1+ externalities in the last 12 months. (These cut points were necessary to have a meaningful split on the dichotomous dependent variable.) As shown in the analysis summary given in Table 3, when Lifetime Drinking Status was controlled, people aged 30–49 and older than 50 were more likely to report 2+ lifetime externalities than those under 30, presumably partly because of longer lives. Compared with whites, Hispanics were less likely to report others’ drinking problems. Married people also had lower risk of harms from others’ drinking. The individual’s lifetime drinking pattern, involving teens, 20s, 30s and current monthly heavy drinking occasions and a history of respondents’ own drinking problems, strongly predicted likelihood of lifetime externalities. Compared with lifetime abstainers, those drinking at least 5+ monthly in their life (but no drinking problems) had odds of 2.4 times to also have 2+ externalities, while the problem drinkers’ odds of ever having at least two externalities, other predictors accounted for, was over 12 times the lifetime abstainers’. Similarly, the respondents’ own current drinking pattern and problem drinking (last 12 months) strongly predicted the likelihood of experiencing at least one externality of the six in the past year. Those drinking 5+ at least monthly (without alcohol problems) and those reporting alcohol problems last year were both significantly more likely to report an externality in the last year than lifetime abstainers, with odds ratios of about 3 and 8, respectively (see Table 3).

In order to further assess how respondents’ own drinking patterns were associated with externalites, segmentation analysis was perform using the CHAID algorithms to investigate whether the sample population may be empirically segregated in terms of their likelihood of experiencing 1+ externalities in the last 12 months by their alcohol consumption volume and the three heavy drinking pattern variables (frequencies of drinking 5+, 8+ and 12+ drinks in a day). First, excluding current abstainers, the 9-level categorized alcohol volume variable (which ranged from <1 drink/month to >4 drinks/day), was chosen as the initial segmentation variable. The CHAID program estimates the rates of reporting one or more externalities within each of the volume categories and then statistically compares these rates amongst all neighboring volume groups. If one or more of these tests results in non-significant differences in outcome rates, CHAID merges the two adjacent volume categories (thus combining individuals in both groups into a volume group with a wider range). This process is repeated until outcome rates in all final adjacent volume groups differ significantly. The process resulted in selection of five empirically distinct volume groups each differing in externality prevalence. Then, within each resultant volume category, the program chose among available pattern variables (the heavy drinking frequencies of 5+, 8+, and 12+) selecting first the pattern variable and cut points that yielded the most significant discrimination of externality prevalence.

Table 4 summarizes the volume and heavy drinking pattern categorizations that resulted from this segmentation analysis (including additionally data on lifetime abstainers and ex-drinkers). Out of five resultant volume categories, the highest three were further split by 5+ or 12+ frequencies (as dichotomies). As the analysis was performed among those who drank alcohol in the last 12 months, separately derived findings for lifetime abstainers and ex-drinkers are also presented in the table for comparison. As shown in Table 4, among abstainers, ex-drinkers and light drinkers (2 drinks/month or less), drinking status did not appear to be strongly associated with 1+ externalities (although fine-grain empirical differences were obtained). For the three highest volume levels, both volume and heavy drinking pattern proved influential and positively associated with externalities. Furthermore, as given for comparison in the table, volume and pattern were also associated with the respondents’ own drinking problems (the latter results are derived independently from the CHAID analysis).

Finally, the association between others’ and one’s own drinking problems may be gauged in a summary fashion in Table 5 by the correlations between 1+ or 2+ externalities and four drinking problem measures (1+ or 2+ consequences, 3+ DSM-IV dependence and 1+ life-area harms of a list of six).

Significant correlations were found between all measures, with 1+ consequence and 1+ life harms tending to have larger association with externality measures. It should be noted that two of the life area harm items––harms from one’s own drinking to home life or marriage and to one’s financial position––parallel two of the externality items. In a subset of approximately 600 cases where balloting was not done, all black non-Hispanic, Hispanic and Other’ (e.g., Asian American, American Indian or Native Alaskan) ethnic group cases, an item assessing the respondent’s sense of how easy it was to buy alcohol in the evening in their neighborhood was available. The hypothesis that alcohol availability would be positively associated with exposure to externalities was confirmed by a Chi-square test (11.12, df = 1) which indicated that ever having two or more lifetime harms from others’ drinking was significantly associated (*p* = 0.001) with local ease of purchase (results not shown). The 12-month 1+ externality indicator was not significantly related to perceived evening availability; although the direction of influence was correct, the base rate and small sample suggest lower power in this instance.

## 4. Discussion

Three reasons might be given for considering externalities important. The first is their extent and cost to society, which requires further consideration and research. Pogue and Sgontz [1], it will be recalled, estimated 22.4% of the total US alcohol abuse costs in 1983 to be external, but improved and current estimates are needed. The second reason is that externalities are unevenly distributed and equity considerations imply cost shifting, which suggests the need to reconsider optimal taxation policies. The third reason is that increasing the public, government regulator and policymaker awareness of externalities might help secure or maintain a more balanced regulatory framework surrounding alcohol, given that various commercial and trade globalization forces seem to press toward an erosion of alcohol controls.

Aside from the standard caveats that apply to all such self-reported experiences and use of telephone survey methods, an additional limitation in this research springs from the respondent’s attribution of the harms from others as being due to these others’ drinking problems. One does not know how accurate this attribution is but, from one public policy viewpoint, this may be less of a difficulty since such perceptions potentially may affect behavior and public policy opinions [32], including the belief that alcohol control measures should be strengthened [35]. On the one hand, there may be some denial or simply forgetting of instances where one was deleteriously affected by others’ drinking; on the other, offsetting this may be an over-interpretation of the occurrence of drinking by the other as a cause of the negative event (which could have occurred independent of the alcohol consumption). Such factors, in opposite directions, may make the causal connection difficult to estimate with precision from self-reports. Another methodological issue relates to the span of the set of questions designed to tap the effects of others’ drinking on the respondent. As noted in the Introduction, some three quarters (78%) of Canadians had experienced at least one of 10 problems from someone else’s drinking in their lifetime and 45% during the prior year. Rates found here (Table 2) of 60% and 9% respectively, are lower, but so too was the number of items (six rather than 10). Thus, the ‘coverage’ of the externalities domain, or number of items assessing it, is an important consideration. Two Canadian items omitted in the present US study were being insulted/humiliated and disturbed by loud parties, which in Canada were most commonly reported, so the different rates of reporting any externality in the two surveys is not surprising. In the US, we did not have the Australian ‘put in fear’ attribution. Because of the pressure for space in most surveys, developing a parsimonious standard set of externalities items is an important agenda. We omitted the additional Canadian items because, though drunken parties and insults may certainly bother others, they may be harder to associate with economic costs than some of the more severe items. Nevertheless, cost estimates for even more severe items may be difficult to estimate. So, from a public policy viewpoint, a remaining agenda is to better characterize the severity and social costs involved in various externalities of drinking. A family whose alcoholic breadwinner loses his or her job may be in serious financial trouble, particularly where jobs are scarce. On the other hand, such a problem could, in good economic times, be more transitory. Similarly, family or marital problems due to another’s (and/or one’s own) drinking may be of any severity and duration, may affect few or many children, and may or may not engage social agencies, with varying implied costs. What price should one put on being ‘put in fear’ by anther’s drinking behavior? While utility cost models suggest some ways this might be addressed, this is a large- rather than a small-scale research agenda.

Methodological research aimed at better characterizing, for individuals reporting them, the distributions in severity, duration and financial burden (or even emotional cost) of such collateral damage from others’ drinking is therefore needed. For example, this future program of research might be modeled upon the efforts of our group to understand what underlies a reported personal health harm [51,52]. Certainly, the current effort to document US national levels of certain non-lethal harms from others’ drinking problems is a small beginning. More than 15 years after the Canadian national study on which the US questions were based, the current results tend to replicate a number of the bivariate demographic findings found there [53], such as the gender-related findings for example, but extend them by considering multivariate ‘influences’ on risks of experiencing harms from others’ drinking. Despite the possibility of marital and family problems from, mostly, male drinking, overall we find being married is slightly protective of experiencing harms from others’ drinking. It seems possible that partnered people are less likely to frequent the places where at least some externalities are most routinely generated, such as assaults around rowdy bars frequented most often by singles. Naturally, older people tend to have accrued more lifetime experiences of the ills of other people’s alcohol abuse. However, on a 12-month basis, younger people experience more externalities. Clearly one’s own drinking pattern has a lot to do with exposure to these risks on both a lifetime and a 12-month basis. In our study, having ever been a heavy or a problem drinker elevated exposure to collateral harms of drinking. Lifetime abstinence and even having become an ex-drinker was associated with lower levels of problems from others’ drinking, perhaps an insulation effect. However, as with lifetime exposure to externalities, which is associated with ever reporting personal heavy or problem drinking, in the 12 month instance based on the CHAID results, harms from others’ drinking was strongly tied to both current high volume and high quantity per day drinking patterns (and associated with personal alcohol problems as well). Drinking occurs in enclaves with heavy abusive drinkers tending to congregate and so this confluence involving a nexus of harms is expected. Examining the potential influence of contexts (or venues) of drinking [54] on experiencing effects of others’ drinking, especially bar and party venues in which the more harmed and harming individuals may congregate––from Table 4 those likely to be younger, male and heavier drinkers––will be a future agenda with these data. The top three CHAID groups each have more than 75% men and they consume the largest number of drinks on average, from at least twice to five times the volume of any other group, with more heavy drinking occasions. Individuals in these groups apparently bump into and harm one another more frequently.

From an economic point of view, there is some question of which collateral harms should be considered ‘personal’ and which true externalities. Most would agree that homicide of a spouse is an external harm; but are reduced financial circumstances for oneself and one’s family external? In one sense, spouse and children of the heavy drinker are innocent collaterals at risk of damage. In another sense they are closely tied and the spouses together may be choosing to continue to affiliate in a problem drinking unit (children have few choices of this kind and may suffer from the alcohol abuse of parent or parents). We will need to look closely at the drinking of partners and their mutual attributions of whose drinking was to blame––but such reports may be self serving and unreliable [55]. Similarly, the heavy drinking of the victim assaulted by another heavy drinker participates in the relative risk of alcohol for assault [20]. Later analyses should consider whether sensation seeking and risk taking attract heavy drinkers into situations where other drinkers may be expected to harm them.

New data will be needed to fully characterize parameters of the externalities for economic and epidemiological purposes. An example is efforts underway to determine the cost of work days lost due to drinking, based on self-reported alcohol-related absenteeism and estimated salary information [56]. Even at present, aside from extreme consequences like alcohol-related homicide (the dead are not surveyed and cannot say they were bludgeoned to death by a drunk), the present self-report data help quantify the extent, if not the cost, of the harm that spreads out from drinkers into their social surroundings through accidents and abusive relationships. Such harms may be useful in adding to the justification for taxation and availability policies designed to reduce external costs, which “selfish” buyers presumably do not adequately take account of or even ignore when they make their outlays for alcohol (we recognize that especially the young tend to ignore personal risks of intoxication as well). An interesting finding based on a small sample of ethnic minority groups involved in this analysis (and thus begging replication) is the association found between perceived evening alcohol availability and experience of harms from others’ drinking, at least on the lifetime basis. This finding is relevant to setting and enforcing reasonable alcohol outlet densities, and monitoring compliance with ordinances related to curbing blight around such outlets in impacted poor and ethnic minority neighborhoods [57]. It will be important in the future to investigate regional differences in externalities which, though possible in the NAS, is beyond the scope of the present article. In the most recent 2009/10 NAS the externalities items were not balloted, so the larger available sample should permit a better regional analysis of variation in alcohol externalities.

The presentation of externality findings may also help communities and policymakers better understand the full toll and risks of drinking *not* borne by the drinker, and may be useful for local policy development, community mobilization, and even for helping provide a rationale for state alcohol controls such as retention of alcohol monopolies in jurisdictions where they exist. This is because clearly not all costs, particularly external costs are included in the personal decision to purchase alcohol, and this should be recognized in setting prices [5].

In Canada it has become apparent in recent discussions leading up to the development of a national strategy on alcohol, in which industry as well as public health representatives have participated, that while the alcohol industry shuns special commodity excise taxation, many producers may find common cause with public health experts in setting minimum prices for classes of alcoholic beverages. This emerged during a presentation on the national strategy at a recent thematic Kettil Bruun Society symposium on *Population Level Studies on Alcohol Consumption & Harm,* held in Toronto, Ontario, October 1–5, 2006, organized by the fifth author. Minimum alcohol prices tend curb the “bottom feeders” and help avert price wars that may deplete earnings of mainstream producers. But they also reduce quality substitution that generally allows the young and problem drinkers to be less sensitive to tax increases. We know that the effectiveness of tax increases in North America is often quickly eroded by inflation since taxes in these jurisdictions are generally not indexed to the Consumer Price Index (yet recent findings indicate reductions in mortality following two alcohol tax increases in spite of this erosion, which supports the value of even non-indexed tax increases [58]). As used in some Scandinavian countries, state alcohol-sales monopolies offer one mechanism for setting minimum prices, or achieving similar effects through markups, although other alcohol beverage control legislation could also potentially be used to establish minimum prices. External harms from individuals’ drinking could provide a strong justification for such price, taxation and regulatory control measures when more fully documented.

## 5. Conclusions

Work on the self-reported externalities from others’ drinking, although some items of this kind have been intermittently included in North American surveys for 20 years, is in its infancy; many conceptual and methodological issues remain to be resolved. Nonetheless, advances in this area, if made, would be of considerable importance to alcohol policy on local, state and national levels. Locally, such issues as neighborhood blight lend themselves to enforcement of existing zoning regulations and nuisance ordinances, and the enactment of stronger ordinance by cities. At the state level, similarly, in many US jurisdictions limits are set on outlet densities, decisions are made regarding alcohol control (for example 18 states, and Montgomery County, Maryland, have wholesale monopolies on spirits, with 12 of these having also retail monopolies). At the national level, too, the economic costs of externalities mount up. Therefore, the careful documentation of economic impacts including, for example, losses in productivity and increases in health costs attributable to alcohol may be compelling to legislators who tend otherwise to think of economic benefits of increased sales, such as increased revenues. In future work the effect of various regulations on reducing externalities (self-reported and assessed through archives) needs to be examined in much greater detail, in collaboration with economists. Measures which raise the floor price for various types of alcoholic beverages are an attractive possibility because these might show greater effects on the heaviest drinkers than broad alcohol tax measures and so have potential for reducing externalities. It remains an important agenda, too, to develop better strategies for linking economic costs to reported externalities. This promises to be an exciting area of environmental research on alcohol, of the highest public health relevance.

## Figures and Tables

**Table 1.** Demographic characteristics of the analytic sample (*N* = 2,550).

**Demographic Characteristic**	**Unweighted *n***	**Weighted Percent**
Male	1,161	51.7
Age: 18–29	497	20.9
30–49	1,005	41.7
50+	991	37.4
Ethnicity: Whites	1,082	74.3
Blacks	826	11.4
Hispanics	511	8.0
Others	131	6.3
Employed (full or part time)	1,561	65.7
Married (or “living with”)	1,254	62.3
Education: High school or less	1,224	39.3
Some college	619	26.5
College graduate +	689	34.1
Family income: < $20,000	742	18.0
$20,000–60,000	873	37.4
≥$60,000	577	31.1
missing	358	13.5
Children aged under 18 in home	1,081	41.4

**Table 2.** Prevalence of Specific Harms from Others’ Drinking and 1+ and 2+ Externalities Indicators in the US General Population (Weighted Sample; *N* = 2,550).

**Item Content**	**Ever %**	**Last 12 Months %**
Been a passenger with a driver who had too much to drink?	44.2	3.3
Been pushed, hit, or assaulted by someone who had been drinking?	28.3	2.4
Had family problems or marriage difficulties due to someone else’s drinking?	17.9	3.4
Had your property vandalized by someone who had been drinking?	12.0	1.8
Been in a motor vehicle accident because of someone else’s drinking?	8.1	0.3
Had financial trouble because of someone else’s drinking?	7.1	1.0

1+ Externalities Indicator	59.6	9.1
2+ Externalities Indicator	34.0	2.0

**Table 3.** Odds Ratios and 95% CIs of Logistic Regressions Predicting Two or More (2+) Externalities Ever and One or More (1+) Externalities in the Past 12-Months.

	**2+ Externalities Lifetime**	**1+ Externalities Last-12-Months**
Male	0.97 (0.72, 1.30)	0.88 (0.56, 1.37)
Age (reference 18–29):		
30–49	1.92 (1.25, 2.94)**	0.70 (0.40, 1.23)
50+	1.77 (1.11, 2.82)*	0.41 (0.21, 0.83)*
Ethnicity (reference whites) :		
Blacks	0.75 (0.51, 1.09)	1.06 (0.62, 1.83)
Hispanics	0.68 (0.49, 0.95)*	1.08 (0.64, 1.83)
Others	1.00 (0.59, 1.70)	0.78 (0.35, 1.74)
Employed (full or part time)	1.12 (0.82, 1.53)	1.20 (0.73, 1.97)
Married or living with	0.73 (0.54, 0.99)*	0.83 (0.53, 1.31)
Education (reference High School or less):		
Some College	1.02 (0.74, 1.41)	1.25 (0.79, 2.00)
College Graduate or higher	0.85 (0.60, 1.21)	0.74 (0.41, 1.31)
Family Income (reference < $20,000):		
$20,000–60,000	0.96 (0.66, 1.39)	0.74 (0.42, 1.31)
$60,000	1.37 (0.90, 2.07)	0.98 (0.53, 1.80)
Missing	0.67 (0.41, 1.07)	0.53 (0.23, 1.25)
Living with Children aged under 18	1.22 (0.88, 1.68).	0.90 (0.53, 1.52)

Drinking characteristics (ref Lifetime Abstainers)		
Ex-drinkers	NA	0.66 (0.26, 1.64)
Never 5+ monthly (lifetime or12-month) a	1.41 (0.87, 2.29)	0.87 (0.37, 2.02)
5+ at least monthly (lifetime or12-month) a	2.43 (1.46, 4.04)**	2.98 (1.16, 7.62)*
Problem Drinker (lifetime or12-month) a,b	12.5 (7.5, 21.1)***	7.59 (2.86, 20.1)***

* p < 0.05

** p < 0.01

*** p < 0.001

a Lifetime is during teens, 20s 30s, as relevant given age (or for Problem Drinker “ever”)

b Reference for Problem Drinker is not reporting problems

**Table 4.** Volume and Heavy Drinking Pattern Classification from CHAID Analysis with Corresponding Rates of Having One or More (1+) Externalities (last 12-months), Showing Characteristics (Gender, Age and Drinking) of Resultant Groups (Weighted Results).

**Volume Group (drinks/time)**	**Heavy Drinking Pattern**	***n***	**Men**	**Age**	**Drinks/year**	**Alcohol Problem**	**1+ Externality**
Abstainers a		517	40%	44	NA	NA	8%
Ex-drinkers a		532	50%	47	NA	NA	5%
1/month or less (but >0)	b	370	41%	44	4	0.8%	9%
1+/month to 2/month	b	163	37%	48	18	0.4%	2%
2+/month to 2/week	Never 5+ 5+ at least once/year	229 62	45% 63%	47 40	61 63	2% 9%	4% 12%
2+/week to 4/day	Never 5+/month 5+ at least monthly	420 170	59% 79%	46 35	365 663	3% 16%	9% 18%
Over 4/day	Never 12+ monthly 12+ at least monthly	36 30	77% 96%	43 33	2123 2621	33% 41%	26% 73%

a Results not from CHAID

b Heavy drinking pattern unimportant in predicting externalities in lowest two volume groups

**Table 5.** Pearson Correlation between Externality Indicators and Alcohol Problems––Both in Previous 12 Months.

	**1+ Externalities**	**2+ Externalities**
1+ Consequences	0.28***	0.14***
2+ Consequences	0.23***	0.10***
3+ DSM-IV Dependence	0.18***	0.16***
1+ Life Area Harms	0.29***	0.15***

*** *p* < 0.001
